# COVID-19 Mortality Prediction From Deep Learning in a Large Multistate Electronic Health Record and Laboratory Information System Data Set: Algorithm Development and Validation

**DOI:** 10.2196/30157

**Published:** 2021-09-28

**Authors:** Saranya Sankaranarayanan, Jagadheshwar Balan, Jesse R Walsh, Yanhong Wu, Sara Minnich, Amy Piazza, Collin Osborne, Gavin R Oliver, Jessica Lesko, Kathy L Bates, Kia Khezeli, Darci R Block, Margaret DiGuardo, Justin Kreuter, John C O’Horo, John Kalantari, Eric W Klee, Mohamed E Salama, Benjamin Kipp, William G Morice, Garrett Jenkinson

**Affiliations:** 1 Mayo Clinic Rochester, MN United States

**Keywords:** COVID-19, mortality, prediction, recurrent neural networks, missing data, time series, deep learning, machine learning, neural network, electronic health record, EHR, algorithm, development, validation

## Abstract

**Background:**

COVID-19 is caused by the SARS-CoV-2 virus and has strikingly heterogeneous clinical manifestations, with most individuals contracting mild disease but a substantial minority experiencing fulminant cardiopulmonary symptoms or death. The clinical covariates and the laboratory tests performed on a patient provide robust statistics to guide clinical treatment. Deep learning approaches on a data set of this nature enable patient stratification and provide methods to guide clinical treatment.

**Objective:**

Here, we report on the development and prospective validation of a state-of-the-art machine learning model to provide mortality prediction shortly after confirmation of SARS-CoV-2 infection in the Mayo Clinic patient population.

**Methods:**

We retrospectively constructed one of the largest reported and most geographically diverse laboratory information system and electronic health record of COVID-19 data sets in the published literature, which included 11,807 patients residing in 41 states of the United States of America and treated at medical sites across 5 states in 3 time zones. Traditional machine learning models were evaluated independently as well as in a stacked learner approach by using AutoGluon, and various recurrent neural network architectures were considered. The traditional machine learning models were implemented using the AutoGluon-Tabular framework, whereas the recurrent neural networks utilized the TensorFlow Keras framework. We trained these models to operate solely using routine laboratory measurements and clinical covariates available within 72 hours of a patient’s first positive COVID-19 nucleic acid test result.

**Results:**

The GRU-D recurrent neural network achieved peak cross-validation performance with 0.938 (SE 0.004) as the area under the receiver operating characteristic (AUROC) curve. This model retained strong performance by reducing the follow-up time to 12 hours (0.916 [SE 0.005] AUROC), and the leave-one-out feature importance analysis indicated that the most independently valuable features were age, Charlson comorbidity index, minimum oxygen saturation, fibrinogen level, and serum iron level. In the prospective testing cohort, this model provided an AUROC of 0.901 and a statistically significant difference in survival (*P*<.001, hazard ratio for those predicted to survive, 95% CI 0.043-0.106).

**Conclusions:**

Our deep learning approach using GRU-D provides an alert system to flag mortality for COVID-19–positive patients by using clinical covariates and laboratory values within a 72-hour window after the first positive nucleic acid test result.

## Introduction

COVID-19 is caused by the SARS-CoV-2 virus and is suspected to be of zoonotic origin, with spillover from bats or pangolins into humans in Wuhan, China [1,2]. COVID-19 has become one of the largest public health emergencies of the past century with over 203 million confirmed cases and 4.3 million deaths as of August 2021 according to the World Health Organization [3]. The COVID-19 pandemic has overwhelmed global medical supply chains, hospitals, and economies, which has led governments to respond with varying policies, including mask mandates and travel restrictions [4,5]. At times, hospitals and health care workers have become so overburdened with patients with COVID-19 that they have been forced to ration care, raising logistical and ethical concerns [6].

The clinical course of COVID-19 is diverse with most individuals experiencing mild or asymptomatic disease, but many patients develop life-threatening diseases, including features such as cytokine storms, thrombotic complications, or severe acute respiratory syndrome requiring mechanical ventilation or extracorporeal membrane oxygenation [7]. A major medical challenge is therefore to reliably triage patients according to their risk for severe disease. Age is consistently observed to be a predominant risk factor for severe disease [7], but deaths are not limited to older adults and the majority of older patients survive COVID-19 [7]. Other comorbidities and laboratory test values are expected to be capable of further individualizing and enhancing mortality prediction. Recent studies investigating statistical and machine learning (ML) models for mortality prediction have confirmed that detailed evaluation of medical records can facilitate further stratification of patients [8-12].

A systematic review of 147 published or preprint prediction models found consistent problems with inherent biases in the data sets investigated or created in all such studies, ultimately concluding that “we do not recommend any of these reported prediction models for use in current practice” [12]. Clinical practices differ in the nature of their observational electronic health record (EHR) data set, patient population, clinical practices, and electronic record or laboratory ordering practices. Correspondingly, the literature review conducted at the outset of this study indicated that the existing prediction models were likely unsuited to our clinical setting without essentially starting afresh by retraining, validating, and testing predictions.

We describe Mayo Clinic’s experience assembling, what is to our knowledge, the largest reported COVID-19 database for mortality prediction and using this database to create a system for COVID-19 mortality prediction, tailored to a unique patient population. Despite the biases inherent to it, because this large and growing database represents a health care system spanning 5 states and 3 time zones over a study window greater than 11 months, our model is expected to be the least confounded and the most generalized COVID-19 mortality predictor published to date. We report the successful development and validation of a state-of-the-art ML model to provide mortality prediction shortly after confirmation of SARS-CoV-2 infection in this Mayo Clinic patient population and discuss in detail the various logistical and scientific challenges involved in the early deployment of such a system in a rapidly changing pandemic environment.

## Methods

### Study Design

This work required the development of a data set and the subsequent modeling of the resultant cohort. After data collection and cleaning, 2 broad classes of algorithms were considered to model this data. The first approach ignores the time series nature of the underlying data and applies traditional ML classifiers. The second approach explicitly models the time series data while dealing with the missing-not-at-random (MNAR) values by using specialized recurrent neural networks (RNNs). Both types of modeling methods were run independently and compared using cross-validation, and a single winning model was selected for prospective performance validation.

### EHR and Laboratory Information System Observational Cohort Data Collection

This study adheres to a research protocol approved by the Mayo Clinic Institutional Review Board. Data were retrospectively collected after March 1, 2020 on COVID-19–positive individuals presenting to a Mayo Clinic site or health system, while excluding patients without research consent or from European Union countries covered by the general data protection regulation law. We restricted our focus to 11,807 patients with a positive COVID-19 nucleic acid test result on or before January 27, 2021 and at least one non-COVID test result. Although the data collection system is deployed and ongoing, the January cutoff was selected for this study to provide sufficient cohort size while allowing a minimum of 3 weeks of follow-up to accurately establish survival status. Mayo Clinic’s EHR and laboratory information system (LIS) contain data from each of its 3 campuses (Rochester, Minnesota; Jacksonville, Florida; Scottsdale, Arizona) as well as the surrounding health system sites spanning 5 states (MN, IA, WI, FL, and AZ). Although the EHR contains clinically reportable laboratory results, many of these can only be reported within defined ranges, which can result in qualitative text values rather than the raw numeric measurements. Because many ML algorithms typically work better with quantitative rather than qualitative results, we used the LIS to gather such laboratory testing measurements and the EHR to gather the remaining variables. The EHR data were queried from an underlying Db2 database (IBM Corp), and the LIS data were queried from an SQL database (Microsoft).

### Multivariate Time-Series Data With Missingness

The clinical covariates collected were age, sex, height, weight, Charlson comorbidity score, temperature, blood pressure, respiratory rate, oxygen saturation (SpO_2_) levels, and diagnoses of chronic kidney disease or diabetes mellitus. Furthermore, we included laboratory test values from a basic metabolic panel, complete blood counts, and some less routine test results of relevance to COVID-19, as determined by scientific literature and physician collaborators. Table 1 details the features collated into our database. In Multimedia Appendix 1, we provide a detailed breakdown of these clinical covariates and laboratory values in our cohort (Table S1 of Multimedia Appendix 1) as well as the cohort’s geographic distribution (Figure S1 of Multimedia Appendix 1). Differentiating between missing data and absence of a condition is not possible from EHR diagnostic codes, particularly for patients treated in an outpatient setting. Therefore, we focused mainly on the Charlson comorbidity index [13], which is populated in our EHR when there is a recorded medical history during a “patient encounter” in the EHR. Thus, this variable is available and can be assigned a value corresponding to no comorbidities, which is distinct from missingness in the case of no recorded medical history in the EHR. However, owing to their emphasis within the literature, we also included chronic kidney disease [9] and diabetes mellitus [14] as independent comorbidity variables using their ICD-10 (International Classification of Diseases) codes while acknowledging that these variables conflate missingness with lack of a condition.

Clinical covariates such as pre-existing conditions, height, and weight were sampled infrequently, whereas heart rate and SpO_2_ were recorded every 15 minutes for inpatients in our EHR, and other laboratory tests were intermediate in terms of frequency. Therefore, to deal with these multiscale time series measurements, we used the laboratory measurements as the starting point to define our sampling time points. For the variables of sex, age, weight, height, diabetes mellitus, chronic kidney disease, and Charlson comorbidity index, we encoded these variables to exist at the first time point only; in our top performing RNN models, we observed no difference in performance using this strategy when compared to repeating the observations at each time point. For the frequently observed variables of blood pressure systole, blood pressure diastole, temperature, pulse, respiratory rate, and SpO_2_, we computed the minimum and maximum measurements for each calendar day and appended these to each laboratory time point during those dates; if no laboratory time point existed on a given day, we created a new one at noon using these minimum and maximum values. We considered time points within ±72 hours of each patient’s first positive polymerase chain reaction (PCR) result and performed a sensitivity analysis on the length of the patient follow-up after this positive test result.

**Table 1:** Feature measurements collected.

Abbreviation	Description (units or levels)
sex	Sex (male or female)
age	Age at time of polymerase chain reaction–positive test result (years)
weight	Weight (kg)
height	Height (cm)
PCR	SARS-CoV-2 nucleic acid test (+ or –)
SERO	SARS-CoV-2 serology antibody test (+ or –)
BASAA	Basophil count test (10^9^/L)
EOSAA	Eosinophil count test (10^9^/L)
HCT	Hematocrit test (%)
HGB	Hemoglobin test (g/dL)
LYMAA	Lymphocyte count test (10^9^/L)
MCV	Mean corpuscular volume test (fL)
MONAA	Monocyte count test (10^9^/L)
NEUAA	Neutrophil count test (10^9^/L)
PLTC	Platelet count test (10^9^/L)
RBC	Red blood cell count test (10^12^/L)
RDW	Red cell distribution width test (%)
WBC	White blood cell count test (10^9^/L)
CRP	C-reactive protein test (mg/L)
D-DIMER	D-dimer test (ng/mL)
FERR	Ferritin test (mg/L)
IL6	Interleukin-6 test (pg/mL)
TRPS	Troponin T test (ng/L)
FIBTP	Fibrinogen test (mg/dL)
LD	Lactate dehydrogenase test (U/L)
IRON	Serum iron test (mg/dL)
TIBC	Total iron binding capacity test (mg/dL)
SAT	Percent iron saturation test (%)
TRSFC	Transferrin test (mg/dL)
BUN	Blood urea nitrogen test (mg/dL)
CHL	Chloride test (mmol/L)
GLU	Glucose test (mg/dL)
CALC	Calcium test (mg/dL)
CREA	Creatinine test (mg/dL)
POTA	Potassium test (mmol/L)
ALB	Albumin test (g/dL)
BICA	Bicarbonate test (mmol/L)
SODI	Sodium test (mmol/L)
BILI	Bilirubin test (mg/dL)
BPsystole	Blood pressure systole (mm Hg)
BPdiastole	Blood pressure diastole (mm Hg)
Temp	Temperature (°C)
Pulse	Heart rate (1/min)
Resp	Respiratory rate (1/min)
SpO_2_	Oxygen saturation (%)
Charlson	Charlson comorbidity index (10-year survival probability)
CKD	Chronic kidney disease (+ or –)
DM	Diabetes mellitus (+ or –)

### Time-Flattened ML Models

Time series data were flattened/encoded to a fixed length list of features by carry forward imputation (ie, selection of the most recently observed covariate values), ensuring compatibility with traditional ML models. Specifically, after the data are flattened in this fashion, it forms a tabular prediction task suitable for any canonical supervised classification algorithm. The recently published [15] Python-based automated ML tool AutoGluon-Tabular (v0.2.0) was utilized to enable standardized and reproducible ensemble stacking of many model classes (eg, deep neural networks, LightGBM boosted trees, CatBoost boosted trees, Random Forests, Extremely Randomized Trees, XGBoost, and k-Nearest Neighbors).

AutoGluon-Tabular models were fit to our tabular data frames using the “AutoGluon.TabularPrediction.fit” function using all the default parameters except eval_metric='roc_auc'. After running the fit function, access to each individual model created by AutoGluon was achieved by the “get_model_names” method on the resulting prediction object. This then allowed us to pass the specified model to the “predict_proba” method’s optional “model” argument for each of the following model types: KNeighborsUnif, KNeighborsDist, NeuralNetFastAI, LightGBMLarge, NeuralNetMXNet, RandomForestGini, ExtraTreesGini, RandomForestEntr, ExtraTreesEntr, LightGBM, XGBoost, LightGBMXT, CatBoost, WeightedEnsemble_L2. Hereon, we refer to WeightedEnsemble_L2 as the “AutoGluon” model since this was the output of the “predict_proba” method when no single model type was specified.

For relatively static features such as height, weight, or Charlson comorbidity index, we would expect the time-flattened models to be at no disadvantage, whereas the more frequently measured data such as laboratory values or blood pressure will lose information, particularly about trends in the covariates. For instance, 2 individuals with a fever of 39°C recorded in the most recent observation would be treated the same even if one had a sustained high fever and the other had a brief downward trending spike. Of course, there are many potential degrees of freedom to capture more information in the flattened data; one could define a fixed number of the most recent observations or fit a line through the observations over time and pass the slope and intercept as features to the classifier. However, ultimately, the choice to flatten the time series is a choice of convenience and one that attempts to leverage the extensive research efforts devoted to tabular prediction, and therefore, we study here only the last observation carried forward modeling, since proper modeling efforts should account for the time series structure in the EHR data. We next look at models of this form.

### RNN Time Series Models

As the second approach, we implemented the modified gated recurrent unit (GRU) binary classification models proposed by Che et al [16] that are capable of accounting for the MNAR patterns within EHR data, and we adopt their notation. For a given patient, we have *D* = 54 variables and a given time series of *T* time points can be represented as a *T* × *D* matrix *X* whose rows *x_t_* ∈ 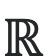
*D*, *t*= 1, . . . , *T* represent the *t*-th observation with *D* variables 
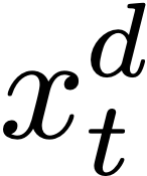
, *d* = 1, . . . , *D*. Accompanying each observation *x_t_* is a time stamp *s_t_* ∈ 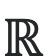, which starts at time 0, *s_1_* = 0 and a binary masking vector *m_t_* ∈ {0, 1}*^D^* with 
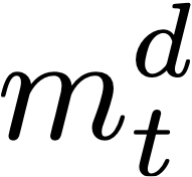
 taking value 1 when 
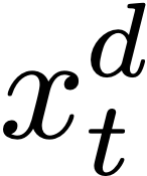
 is observed and 0 otherwise. From these values, we can compute the time intervals.


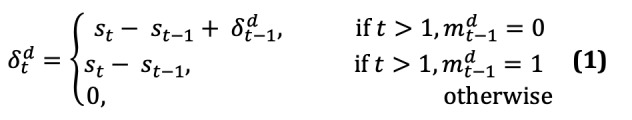


With these definitions, we can look at various modifications to the standard GRU architecture whose *j*-th hidden unit has a reset gate 
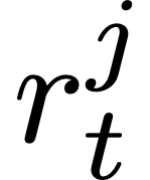
 and update gate 
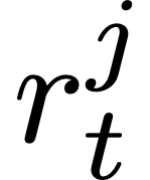
 with hidden state 
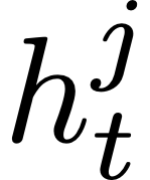
 at time *t* and update the equations.


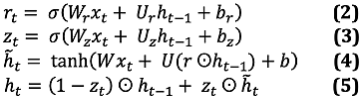


With matrices *W_z_*, *W_r_*, *W*, *U_z_*, *U_r_,* *U* and vectors *b_z_, b_r_, b* composed of model parameters, ⊙ is the Hadamard product, and *σ*(·) is the elementwise sigmoid function. Before modifying the architecture, there are 3 methods to use the GRU above to handle missing data: in “GRU-Mean,” missing values are imputed by their means in the training data; in “GRU-Forward,” missing values are imputed by their last observed value; and in “GRU-Simple,” we simply concatenate the *x_t_*, *m_t_*, and *δ_t_* variables into a single observation vector *x ‘t* and pass this through the GRU equations above. The GRU-D method uses trainable decay weights.


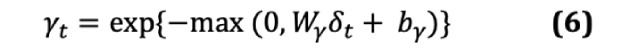


With *W_γ_* and *b_γ_* being trainable model parameters. The observations are then replaced by the update.


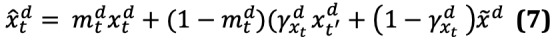


where 
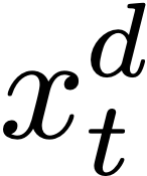
 is the last observed value of the *d*-th variable and 
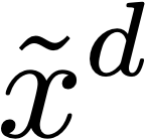
 is the empirical mean of the *d*-th variable in the training data. The modified GRU update equations for GRU-D become the following.


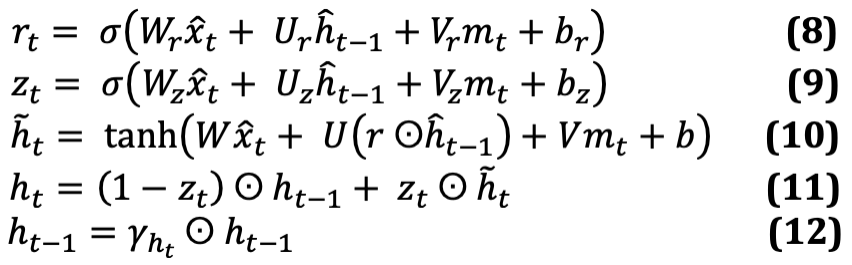


where *V_z_, V_r_, V* are new model parameters to directly handle the masking vector *m_t_* in the model.

Our implementation of the above equations in Python is a slightly modified version of the code available on the GRU-D paper’s [16] GitHub repository. For the core RNN algorithms, we only edited the original GRU-D code, where required, to be compatible with the more recent versions of tensorflow.keras (version 2.1.0) and numpy (version 1.19.2) used in our high-performance computing cluster environment. We selected the specific RNN algorithm by setting the “--model“ argument to be “GRUforward,” “GRU0,” “GRUsimple,” and “GRUD” for GRU-forward, GRU-mean, GRU-simple, and GRU-D, respectively. We utilized the default hyperparameters of the algorithm; however, in our testing, we found that increasing the batch size from 32 to 256 facilitated faster training of the algorithms. Therefore, a batch size of 256 is the only nondefault hyperparameter selection made in our implementation of the RNN algorithms.

### Temporal Cohort Split

**Figure 1:**
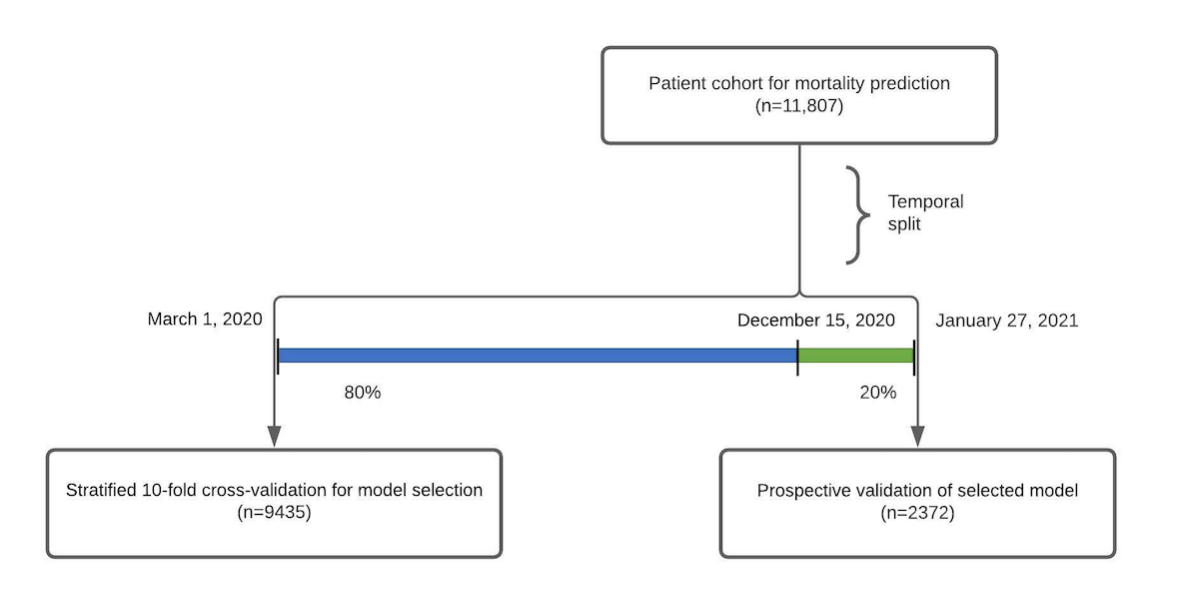
CONSORT (Consolidated Standards of Reporting Trials) diagram demonstrating the temporal split of our cohort for model selection and prospective validation.

As depicted in the CONSORT (Consolidated Standards of Reporting Trials) diagram of Figure 1, patients who first tested positive for COVID-19 from March 1, 2020 through December 15, 2020 (9435/11,807, 79.9%) were assigned to a model selection cohort, whereas patients who first tested positive for COVID-19 from December 16, 2020 through January 27, 2021 (2372/11,807, 20.1%) were used as a prospective testing cohort for the final algorithm. All experiments in the model selection cohort were performed using an identical 10-fold stratified cross-validation using binary classification with the positive class defined as death within 21 days of the first positive PCR test result. Only the single best performing model was evaluated on the prospective cohort after being fit against the entire model selection cohort.

## Results

### Model Selection

**Figure 2:**
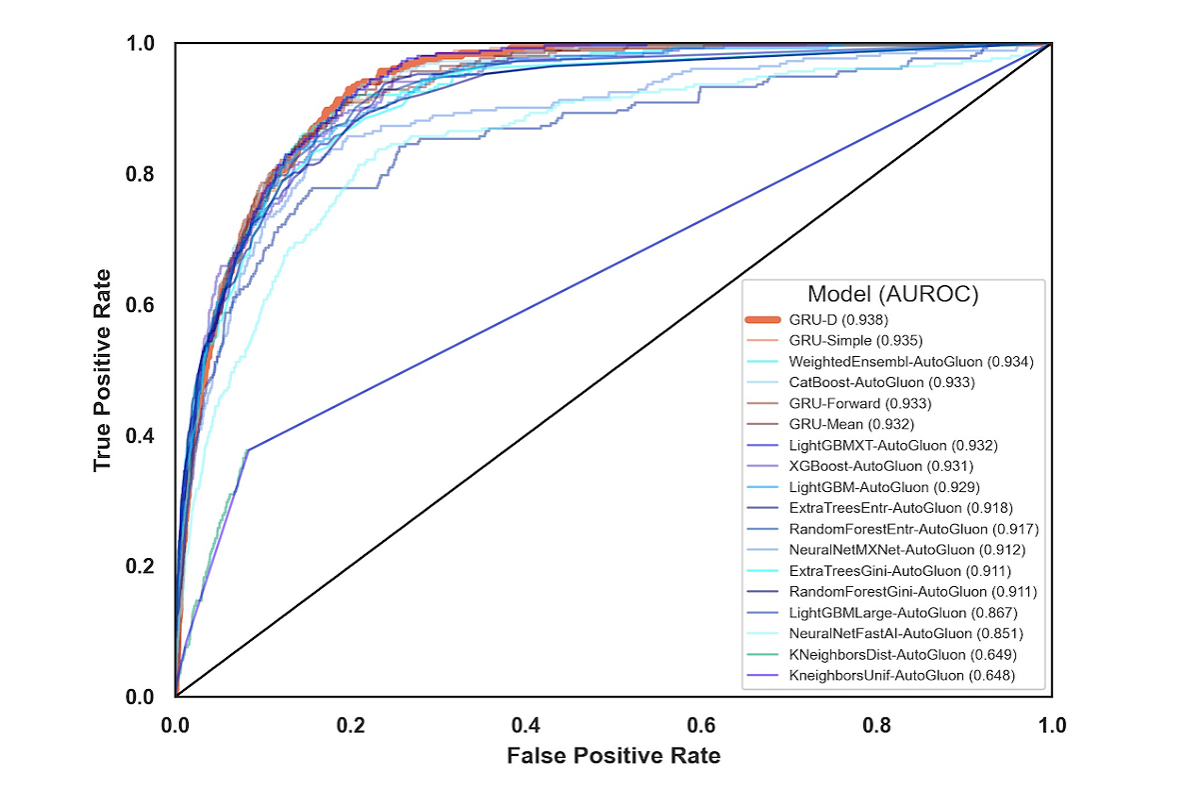
Receiver operating characteristic curves for the 18 models evaluated. AUROC: area under the receiver operating characteristic.

In Figure 2 and Table 2, we compared the results of our various models by using cross-validation area under the receiver operator characteristic (AUROC) curve in the training cohort. Although not in a statistically significant way, we recapitulated the findings of Che et al [16], discovering that the GRU-D model has the highest average cross-validation AUROC curve among all other standard variants of GRU modeling in time series with missing values. In addition, GRU-Simple has higher average cross-validation AUROC curve than the GRU-Forward and GRU-Mean, and the most notable difference underlying these categories is the inclusion of missingness indicators as features to GRU-Simple, which could indicate the value of MNAR patterns in the classification task. GRU-D’s biologically inspired architecture attempts to make even more efficient use of this information and exceeds the performance of all the tested RNN methods. AutoGluon, which only had access to the last measurement of each variable, showed strong performance despite this limitation. In Table 2, each individual AutoGluon model was also benchmarked (those with suffix “-AutoGluon”), along with the final ensemble estimate (labeled simply as “AutoGluon”). Although GRU-D ultimately outranked AutoGluon, each method’s performance fell within the other’s standard error intervals. AutoGluon’s automated hyperparameter tuning and model stacking may indicate that GRU-D could benefit from the addition of hyperparameter search. However, this process may risk overfitting this cross-validation data set, and thus, we selected GRU-D with the default settings rather than attempting to further improve the cross-validation AUROC curve via hyperparameter optimization.

**Table 2:** Modeling results sorted by performance.

Model	Area under the receiver operator characteristic curve (SE)
KNeighborsUnif-AutoGluon	0.648 (0.011)
KNeighborsDist-AutoGluon	0.649 (0.011)
NeuralNetFastAI-AutoGluon	0.858 (0.013)
LightGBMLarge-AutoGluon	0.867 (0.014)
NeuralNetMXNet-AutoGluon	0.907 (0.008)
RandomForestGini-AutoGluon	0.911 (0.007)
ExtraTreesGini-AutoGluon	0.911 (0.009)
RandomForestEntr-AutoGluon	0.917 (0.008)
ExtraTreesEntr-AutoGluon	0.918 (0.007)
LightGBM-AutoGluon	0.929 (0.007)
XGBoost-AutoGluon	0.931 (0.006)
LightGBMXT-AutoGluon	0.931 (0.005)
GRU-Mean	0.932 (0.005)
GRU-Forward	0.933 (0.006)
CatBoost-AutoGluon	0.933 (0.005)
AutoGluon	0.934 (0.005)
GRU-Simple	0.935 (0.004)
GRU-D	0.938 (0.004)

### Length of Time Series

Clearly, we would expect availability of more time series data to result in improved model performance. To determine if predictions could be made utilizing data prior to 72 hours of a patient’s first positive PCR test result, we assessed the performance of GRU-D when we restricted the time series to 12, 24, 48, and 72 hours of follow-up after the first positive PCR test result. The results in Table 3 demonstrate that although we lose performance when predicting earlier in the patient’s disease, we are still able to provide accurate predictions even using data within the same day (12 hours of follow-up) that a patient tests positive for COVID.

**Table 3:** GRU-D performance versus length of time series.

Follow-up after positive finding for polymerase chain reaction	Area under the receiver operator characteristic curve (SE)
12 h	0.916 (0.005)
24 h	0.919 (0.006)
48 h	0.925 (0.005)
72 h	0.938 (0.004)

### MNAR as an Asset and Feature Importance

**Figure 3:**
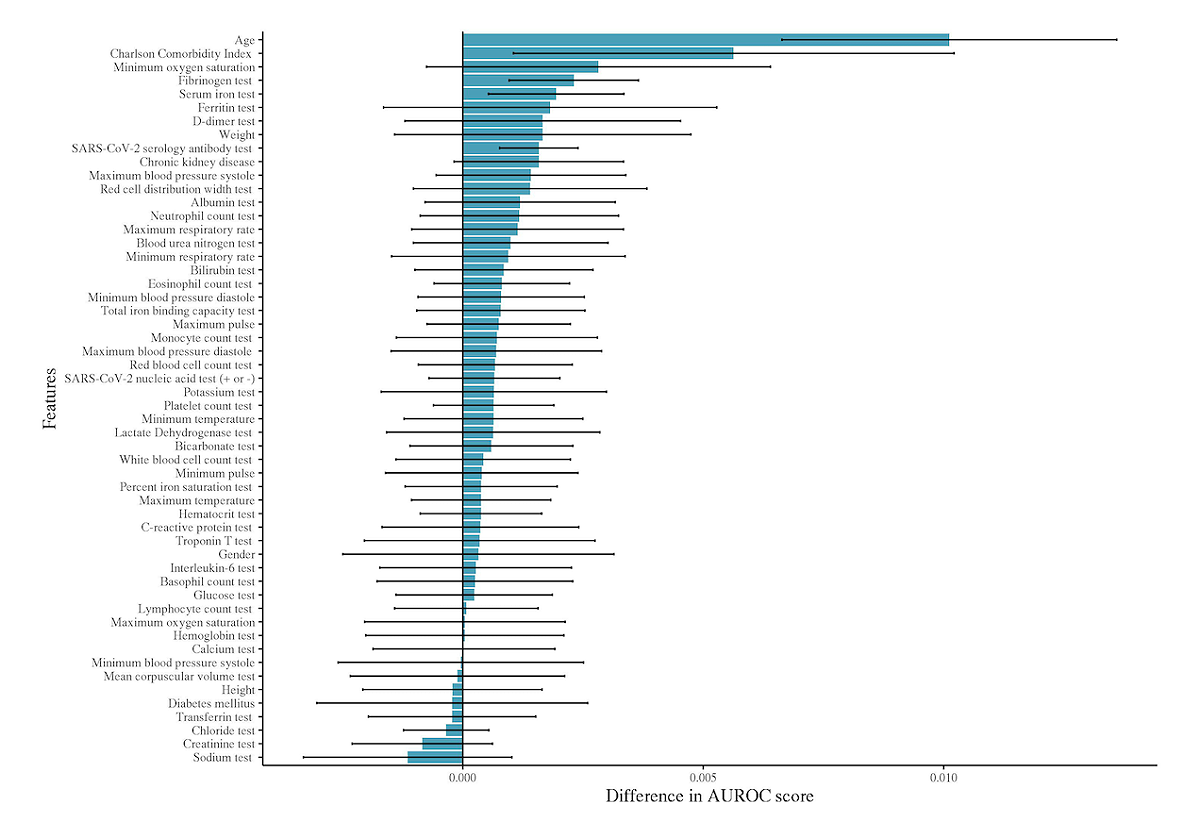
Feature importance in the GRU-D recurrent neural network model as defined by the average drop in the area under the receiver operator characteristic curve (with 95% CI) when each feature is individually removed from the analysis. The top 5 features are seen to be age, Charlson comorbidity index, minimum oxygen saturation, fibrinogen levels, and serum iron levels. AUROC: area under the receiver operator characteristic.

To demonstrate the fact that MNAR data can improve model predictions by GRU-D, we generated a synthetic data set with laboratory test values replaced by Bernoulli coin flips. Therefore, the only valuable information contained within this data set’s laboratory values is the missing data patterns that can be viewed as encoding clinical suspicion or concern. For instance, the D-dimer laboratory value is ordered less frequently than other tests, and therefore, its presence alone can be informative of clinical concern for thrombotic events. Our results found that randomizing the laboratory values resulted in an AUROC curve of 0.904 (0.006), which indicates that the laboratory values in aggregate contributed 0.034 to the AUROC score (since this is the drop in performance compared to the model with actual laboratory values). We ran a further experiment omitting the laboratory values entirely, which produced a lower AUROC of 0.890 (0.006). Therefore, the missing patterns alone contributed 0.014 to the AUROC. To contextualize this finding, we dropped each feature individually from the model, assessed the decrease in the AUROC score, and summarized the top 10 features in the decreasing order of the difference in the AUROC score (Figure 3). We note here that the drop due to missing patterns exceeds the drop due to removing any single variable from the analysis, making the MNAR pattern one of the most valuable pieces of information available to GRU-D. In Multimedia Appendix 1, we show a detailed error analysis of our model using these top 10 features. The fact that age and Charlson comorbidity index are the most significant contributors to mortality prediction is consistent with the well-known risk factors for COVID mortality. The findings of the fibrinogen test, serum iron test, and ferritin test were the 3 most important laboratory values in our models. The presence of chronic kidney disease, weight, serology test, and SpO_2_ were the clinical covariates that also ranked in the top 10 variables by importance. Interestingly, height had low importance, indicating that BMI may not be as effective as weight itself in mortality prediction. However, a limitation of this drop-one-feature variable importance is that a low-ranking feature such as height cannot be said to be irrelevant, just that any information it carries is redundant within other features.

### Prospective Validation and Survival Analysis

To demonstrate the efficacy of our proposed mortality prediction, we performed a Kaplan-Meier analysis using the survival R library [17]. Specifically, we chose a decision boundary on the GRU-D model’s ROC curve, which provided a specific delineation of high-risk and low-risk groups of patients. In our cross-validation cohort, binary classification provides accuracy of 89% (95% CI 88%-90%), recall of 80% (95% CI 74%-85%), precision of 17% (95% CI 15%-19%), and a negative predictive value of 99% (95% CI 99%-100%). Furthermore, although the precision is somewhat low with numerous false positives, we see among the survivors over twice the rate of mechanical ventilation or extracorporeal membrane oxygenation when they are predicted to die by GRU-D (Fisher exact test *P*<.001, odds ratio 2.1, 95% CI 1.8-2.5). We validated this performance in our prospective testing cohort, finding an AUROC of 0.901, accuracy of 78% (95% CI 76%-79%), recall of 85% (95% CI 77%-91%), precision of 14% (95% CI 12%-17%), and a negative predictive value of 99% (95% CI 99%-100%).

**Figure 4:**
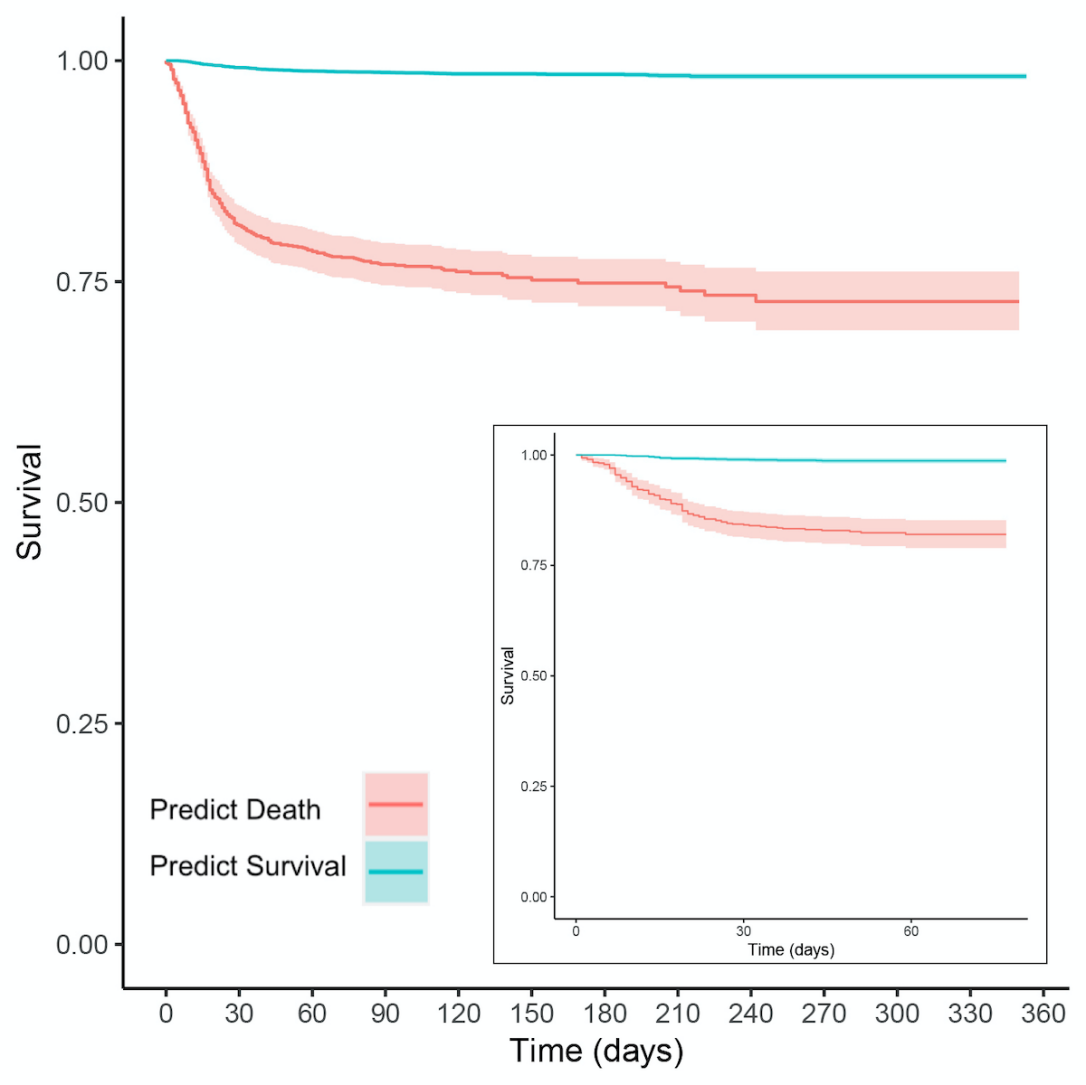
Kaplan-Meier survival curves for the GRU-D stratified populations in the cross-validation cohort (main figure) and the prospective test cohort (inset), where teal is the prediction of low risk of death and red is the prediction of high risk. Both figures have 95% CIs visualized for the teal and red curves, although the teal confidence bands are tight due to our large sample sizes.

Our Kaplan-Meier analysis results in Figure 4 demonstrate the statistically significant stratification provided by our ML model in both the cross-validation and prospective testing experiments. Building a Cox Proportional Hazards model for our prediction in the cross-validation cohort provides a statistically significant difference in survival between the 2 groups (*P*<.001 for the likelihood ratio, logrank, and Wald tests), with a prediction of survival having a substantially improved hazard ratio of 0.053 (95% CI 0.043-0.066). We validated this finding in the prospective testing cohort with a statistically significant difference in survival between the 2 groups (*P*<.001 for the likelihood ratio, logrank, and Wald tests), with a prediction of survival having a substantially improved hazard ratio of 0.067 (95% CI 0.043-0.106).

## Discussion

### Study Overview

In this study, we collected and processed over 50 laboratory and clinical covariates in a population of nearly 12,000 Mayo Clinic patients who tested positive for SARS-CoV-2 by PCR. In this large and geographically diverse data set, we found that the GRU-D RNN could provide state-of-the-art mortality prediction. This performance remained strong even in a held-out test set that mimics how a deployed system would be trained retrospectively and then prospectively utilized in a clinically evolving pandemic setting.

### Principal Results

Our cross-validation experiments summarized in Table 2 indicated that the top performing model to predict mortality in our cohort was the GRU-D RNN. We thus selected the GRU-D method to predict the mortality of patients with COVID-19 and prospectively found an AUROC of 0.901, accuracy of 78% (95% CI 76%-79%), recall of 85% (95% CI 77%-91%), precision of 14% (95% CI 12%-17%), negative predictive value of 99% (95% CI 99%-100%), and a statistically significant difference in survival (*P*<.001, hazard ratio for those predicted to survive, 95% CI 0.043-0.106). As can be expected in prospective validation, we observed a modest drop in AUROC although most of the performance characteristics were close to their original cross-validation estimates, that is, the negative predictive value was largely unchanged, while precision and accuracy showed minor decreases with the recall showing modest improvements.

We chose a prospective/retrospective split in time since this is the most realistic way to assess the potential performance of a system if launched clinically, because it would be trained on data up until its go-live date and then run prospectively in a potentially evolving pandemic environment. Notably, the cutoff date for the 80/20 split creating the prospective test set was December 15, 2020, which is the day after the first COVID vaccine received the United States Food and Drug Administration approval, meaning that our prospective cohort represented a distinctly different clinical environment compared to the period in which the model was trained. The relatively minor loss of performance in prospective validation shows the robustness of the modeling herein, but the observed loss of performance also demonstrates the need for continued retraining/validation of such a model during a constantly evolving pandemic.

### Limitations

The application and deployment of ML methods in clinical practice require concerted care and diligence. One may be inclined to interpret the high negative predictive value of our prediction algorithm as an indication that the best use of the algorithm in practice is as a screening mechanism to discharge patients who are not at risk in order to conserve resources for higher-risk individuals. However, such a conclusion illustrates a pitfall of using a correlative prediction algorithm to make causal conclusions. The algorithm is highly confident that under the current standards of care at Mayo Clinic, these individuals are not likely to succumb to their illness; this is quite distinct from asserting that it is safe to reduce the care for these patients. Arriving at this latter conclusion would likely require a randomized controlled trial, and given the much lower survival rate published in the New York City data set [11] where medical systems were overcapacity, it seems unlikely that reducing care from those who survived in our cohort would have been a safe measure. Because the Mayo Clinic health systems have not been overcapacity, our mortality predictions should be viewed as representing patient stratification when full clinical support is available.

Therefore, we conclude that the algorithm is better deployed as an alert system that flags only those patients it deems as high risk to provide the treating physician with an additional data point that aims to summarize the many covariates and the laboratory values routinely available. In this context, the algorithm has had abundant experience in the provider’s system, effectively “seeing” all patients with COVID-19 that have attended Mayo Clinic and conveying these lessons to physicians who could not have gained such experience personally.

A web interface to this model may allow for widespread usage but given the complexity and error-prone nature of users providing the high dimensional time-series measurements with correct units, the system is better suited for integration within the EHR/LIS infrastructure. We are now exploring the details of deployment of such a GRU-D alert system, which involves discussions with physicians to assess numerous implementation details, for example, deciding whether the alerts would be passive EHR/chart-based flags or a direct page to the frontline clinical provider. Passive chart alerts are less intrusive to existing workflows (ie, a direct page interrupts a physician while tending to other patients) but also provide less-immediate feedback. Additionally, active alerts could also be sent to a triage group to consider if evaluation is needed (for example, from the registered respiratory therapist) rather than interrupting bedside clinicians. Furthermore, for either type of alert, there is the question of prescribing a bedside assessment or leaving it to provider discretion, which is again a matter of balancing disruption of the workflow with the likelihood of missing a critical event. There will not be a universally appropriate implementation for all hospital systems owing to staffing and procedural differences. However, since our algorithm predicts overall COVID-19 mortality and is not tailored to flag imminent events such as cardiopulmonary arrest, it may be appropriate to consider less intrusive chart alerts without prescribed bedside follow-ups.

We have also seen nuances in the challenges and opportunities presented by MNAR data. In the context of traditional statistical inference and imputation, MNAR data is a worst-case scenario so challenging that many practical applications effectively ignore the reality and proceed with algorithms designed for the missing completely at random or missing at random settings. A diligent statistician making this decision may perform a sensitivity analysis under a very limited set of assumed MNAR mechanisms to provide some assurances regarding the robustness of the chosen imputation or analytical strategy [18]. However, here we have demonstrated that classification problems can be quite distinct in this regard. Specifically, if the missing data mechanism is tightly coupled to the ultimate prediction task, it is entirely possible for MNAR data to be an asset rather than an impediment. One can construct a context where the class label is so tightly linked to the missing data mechanism that the patterns of missingness provide more discriminative power than the underlying values themselves (see Multimedia Appendix 2) [19]. In LIS systems, the number of potential laboratory tests that could be ordered at any time is astronomical, and it is unlikely that a practicing physician will ever order a “complete observation” of every test available on a single patient at every point in time. Instead, tests are ordered based on reasonable clinical suspicion that a test might return an abnormal result. From a prognostication point of view, this clinical suspicion is an enormously valuable piece of information that will almost never be captured in a structured data field in the EHR. If an algorithm cannot build off of this clinical suspicion as a starting point, it is also likely that its conclusions may appear to be a “step behind” the ordering clinician. Instead, an algorithm should learn what it can from the MNAR data patterns (here partly encoding clinical suspicion) in addition to the final value returned by the laboratory test.

We also note some of the real-world challenges that are faced when attempting to deploy such an alert system into clinical practice. First, in the retrospective experimental design followed here and by other papers in the literature, the time series data are constructed using the time of sample collection since this is the most biologically accurate way to represent the data and build predictive models. However, in practice, if there can be delays in the turnaround for certain tests, this will either result in delayed predictions (so that the deployed testing data match its retrospective training counterpart) or result in biased predictions when delayed laboratory test results are treated as missing. Therefore, although 72 hours is early in the course of illness, it is crucial that we have demonstrated reasonable performance even when only considering data collected on the same day as the first positive PCR result, because a real-world delay of 48 hours on certain laboratory test values may occur during a global pandemic, and thus, it is critical that the system can still provide accurate and timely predictions even when laboratory test results are delayed. Additionally, with vaccines now being delivered, the models presented herein should be considered as mortality predictions for an unvaccinated individual, and in practice, a vaccinated individual will be expected to be at low risk for mortality based on the clinical trials data.

Another challenge in dealing with LIS data comes from nonstandardization of test coding prior to reporting to the EHR. In a multisite system, the same laboratory test may have multiple test codes to account for the different ordering facilities or variability in local billing regulations. This creates the potential for discrepancies in the values stored within the underlying database such as differing units of measure. Substantial effort is therefore devoted to linking the LIS results to the EHR to ensure consistency across test codes and complete coverage of results in the EHR. The COVID-19 pandemic has created added complexity due to the rapidly evolving and continuously updating availability of COVID-19 nucleic acid and antibody tests. Therefore, effective data collection and deployment of ML methodologies necessitates extensive team-based laboratory and medical expertise to ensure that data aggregation and modeling efforts can be rapidly modified to suit the changing nature of the underlying data set. Scalability also presents practical challenges. This is illustrated by a scenario in which internal workflows began to fail due to limitations in the number of query results being returned by Tableau, necessitating that SQL queries take place on a high-performance computing cluster using a Python/Pandas toolchain. Although these logistical challenges may be of limited academic interest, they are important to document, as such barriers have been a greater impediment to rapid real world deployment than more traditional topics in the ML literature such as the identification of appropriate classification algorithms.

### Comparison With Prior Work

For context, in Table 4, we summarize some of the largest published COVID-19 mortality studies and specifically, the cohorts analyzed and the most relevant features identified. When smaller cohorts see insufficient numbers of deaths for direct mortality prediction, studies tend to focus on the prediction of severe outcomes. For instance, in a cohort of 123 patients with COVID-19 in Vulcan Hill Hospital, China, in the study of Pan et al [20], the mortality classifier based on XGBoost yielded an AUC of 0.86-0.92. Likewise, in a cohort of 372 Chinese cases (99.7% cohort survival rate), Gong et al [9] found that the following variables provided an AUROC of 0.85. Similarly, in a study of 375 patients with COVID-19 conducted by Ko et al [21], the mortality prediction model based on XGBoost had 92% accuracy. In a study of 398 COVID-19–positive patients by Abdulaal et al [22], 86% accuracy was achieved (95% CI 75%-93%). In a large study of 2160 cases over 54 days from 3 hospitals in Wuhan, China with sufficient cases to assess mortality (88% cohort survival rate), Gao et al [8] reported 0.92-0.98 as the AUROC using an ensemble classifier. Furthermore, Vaid et al [11] used 4098 inpatient cases over 68 days in New York City (83% cohort survival rate) to achieve an AUROC of 0.84-0.88 in mortality prediction. Kim et al [23] studied 4787 patients and their XGBoost-based classifier demonstrated an AUC of 0.88-0.89 (95% CI 0.85-0.91) in predicting the need for intensive care, which is distinct from mortality prediction. Bolourani et al [24] studied 11,525 patients to achieve an AUROC of 0.77 in predicting respiratory failure within 48 hours of admission, which is also distinct from mortality prediction, based on data from the emergency department by using an XGBoost model.

The dramatically different cohort mortality rates and the associated predictive accuracies may be in part due to the differing straining of the local health care systems at the time of study (both Wuhan and New York City experienced waves of patients that at different times overwhelmed the health care infrastructure), and the relatively geographically narrow nature of each of these data sets underscores why it is unlikely that these mortality predictions would extend directly to our patient population in a health care system spanning 3 time zones and multiple locales unrepresented in the literature.

**Table 4:** Summary of the related studies.

Study	Patients (n)	Model/algorithm	Cohort survival	Prediction	Area under the receiver operating characteristic curve	Feature importance
Pan et al [20]	123	XGBoost	52.8%	Mortality	0.86-0.92	Lymphocyte percentage, prothrombin time, lactate dehydrogenase, total bilirubin, eosinophil percentage, creatinine, neutrophil percentage, and albumin level
Gong et al [9]	372	Nomogram	99.7%	Severity	0.85 (95% CI 0.790-0.916)	Higher lactate dehydrogenase, C-reactive protein, red blood cell distribution width, direct bilirubin, blood urea nitrogen, and lower albumin
Ko et al [21]	375	XGBoost	98.1%	Mortality	—,^a^ accuracy of 92%	Not assessed
Abdulaal et al [22]	398	Artificial neural network	—	Mortality	—, accuracy of 86% (95% CI 75%-93%)	Altered mentation, dyspnea, age, collapse, gender, and cough
Shi et al [10]	487	Custom risk score calculation	100%	Severity	—	Advanced age, presence of hypertension, and being male
Gao et al [8]	2160	Ensemble model based on logistic regression, gradient-boosted decision tree, neural network, and support vector machine	88%	Mortality	0.92-0.98	Consciousness, chronic kidney disease, lymphocyte counts, sex, sputum, blood urea nitrogen, respiratory rate, oxygen saturation, D-dimer, number of comorbidities, albumin, age, fever, and platelet count
Vaid et al [11]	4098	XGBoost	83%	Mortality	0.84-0.88	Age, anion gap, C-reactive protein, lactate dehydrogenase, oxygen saturation, blood urea nitrogen, ferritin, red cell distribution width, and diastolic blood pressure.
Kim et al [23]	4787	XGBoost	—	Need for intensive care	0.88-0.89	Activities of daily living, age, dyspnea, body temperature, sex, and underlying comorbidities
Bolourani et al [24]	11,525	XGBoost	—	Predicting respiratory failure	0.77	Invasive mode of oxygen delivery being a nonrebreather mask, emergency severity index values of 1 and 3, maximum respiratory rate, maximum, oxygen saturation, Black race, age on admission, eosinophil percentage, serum sodium level, and serum lactate level.
This study	11,807	GRU-D	95.4%	Mortality	0.938 cross-validation; 0.901 prospectively	Figure 2, top 5: age, Charlson comorbidity index, minimum oxygen saturation, fibrinogen level, and serum iron level

^a^Not available.

As indicated in Table 4, this study represents the largest cohort collected for mortality prediction in COVID-19, and the GRU-D algorithm shows state-of-the-art performance. Notably, many papers selected models based on XGBoost, which also showed strong cross-validation performance in our data. However, Table 2 demonstrates that XGBoost was not even in the top 5 algorithms that we assessed. Additionally, in agreement with Gao et al [8], we find that ensemble algorithms such as AutoGluon can provide stronger performance, although as noted previously, the GRU-D algorithm ended up ranked most highly in our cross-validation experiments.

### Conclusions

We have aggregated and analyzed one of the largest multistate COVID-19 EHR databases for mortality prediction. Using this database, we have trained and prospectively validated a highly effective ML algorithm using the GRU-D neural network architecture to predict the mortality of patients with COVID-19 shortly after their first positive PCR test result.

## Appendix

Multimedia Appendix 1 Error analysis.

Multimedia Appendix 2 Monte Carlo missing-not-at-random simulation.

## Abbreviations

AUROC: area under the receiver operator characteristic
EHR: electronic health record
GRU: gated recurrent unit
LIS: laboratory information system
ML: machine learning
MNAR: missing-not-at-random
PCR: polymerase chain reaction
RNN: recurrent neural network

## References

[1] Andersen KG, Rambaut A, Lipkin WI, Holmes EC, Garry RF (2020). The proximal origin of SARS-CoV-2. Nat Med.

[2] Zhou P, Yang X, Wang X, Hu B, Zhang L, Zhang W, Si H, Zhu Y, Li B, Huang C, Chen H, Chen J, Luo Y, Guo H, Jiang R, Liu M, Chen Y, Shen X, Wang X, Zheng X, Zhao K, Chen Q, Deng F, Liu L, Yan B, Zhan F, Wang Y, Xiao G, Shi Z (2020). A pneumonia outbreak associated with a new coronavirus of probable bat origin. Nature.

[3] WHO Coronavirus disease (COVID-19) dashboard. World Health Organization.

[4] Chinazzi M, Davis JT, Ajelli M, Gioannini C, Litvinova M, Merler S, Pastore Y Piontti A, Mu K, Rossi L, Sun K, Viboud C, Xiong X, Yu H, Halloran ME, Longini IM, Vespignani A (2020). The effect of travel restrictions on the spread of the 2019 novel coronavirus (COVID-19) outbreak. Science.

[5] Remuzzi A, Remuzzi G (2020). COVID-19 and Italy: what next?. Lancet.

[6] Rosenbaum L (2020). Facing Covid-19 in Italy - Ethics, Logistics, and Therapeutics on the Epidemic's Front Line. N Engl J Med.

[7] Wiersinga WJ, Rhodes A, Cheng AC, Peacock SJ, Prescott HC (2020). Pathophysiology, Transmission, Diagnosis, and Treatment of Coronavirus Disease 2019 (COVID-19): A Review. JAMA.

[8] Gao Y, Cai G, Fang W, Li H, Wang S, Chen L, Yu Y, Liu D, Xu S, Cui P, Zeng S, Feng X, Yu R, Wang Y, Yuan Y, Jiao X, Chi J, Liu J, Li R, Zheng X, Song C, Jin N, Gong W, Liu X, Huang L, Tian X, Li L, Xing H, Ma D, Li C, Ye F, Gao Q (2020). Machine learning based early warning system enables accurate mortality risk prediction for COVID-19. Nat Commun.

[9] Gong J, Ou J, Qiu X, Jie Y, Chen Y, Yuan L, Cao J, Tan M, Xu W, Zheng F, Shi Y, Hu B (2020). A Tool for Early Prediction of Severe Coronavirus Disease 2019 (COVID-19): A Multicenter Study Using the Risk Nomogram in Wuhan and Guangdong, China. Clin Infect Dis.

[10] Shi Y, Yu X, Zhao H, Wang H, Zhao R, Sheng J (2020). Host susceptibility to severe COVID-19 and establishment of a host risk score: findings of 487 cases outside Wuhan. Crit Care.

[11] Vaid A, Somani S, Russak AJ, De Freitas JK, Chaudhry FF, Paranjpe I, Johnson KW, Lee SJ, Miotto R, Richter F, Zhao S, Beckmann ND, Naik N, Kia A, Timsina P, Lala A, Paranjpe M, Golden E, Danieletto M, Singh M, Meyer D, O'Reilly PF, Huckins L, Kovatch P, Finkelstein J, Freeman RM, Argulian E, Kasarskis A, Percha B, Aberg JA, Bagiella E, Horowitz CR, Murphy B, Nestler EJ, Schadt EE, Cho JH, Cordon-Cardo C, Fuster V, Charney DS, Reich DL, Bottinger EP, Levin MA, Narula J, Fayad ZA, Just AC, Charney AW, Nadkarni GN, Glicksberg BS (2020). Machine Learning to Predict Mortality and Critical Events in a Cohort of Patients With COVID-19 in New York City: Model Development and Validation. J Med Internet Res.

[12] Wynants L, Van Calster B, Collins GS, Riley RD, Heinze G, Schuit E, Bonten MMJ, Dahly Darren L, Damen Johanna A A, Debray Thomas P A, de Jong Valentijn M T, De Vos Maarten, Dhiman Paul, Haller Maria C, Harhay Michael O, Henckaerts Liesbet, Heus Pauline, Kammer Michael, Kreuzberger Nina, Lohmann Anna, Luijken Kim, Ma Jie, Martin Glen P, McLernon David J, Andaur Navarro Constanza L, Reitsma Johannes B, Sergeant Jamie C, Shi Chunhu, Skoetz Nicole, Smits Luc J M, Snell Kym I E, Sperrin Matthew, Spijker René, Steyerberg Ewout W, Takada Toshihiko, Tzoulaki Ioanna, van Kuijk Sander M J, van Bussel Bas, van der Horst Iwan C C, van Royen Florien S, Verbakel Jan Y, Wallisch Christine, Wilkinson Jack, Wolff Robert, Hooft Lotty, Moons Karel G M, van Smeden Maarten (2020). Prediction models for diagnosis and prognosis of covid-19: systematic review and critical appraisal. BMJ.

[13] Charlson ME, Pompei P, Ales KL, MacKenzie CR (1987). A new method of classifying prognostic comorbidity in longitudinal studies: development and validation. J Chronic Dis.

[14] Kumar A, Arora A, Sharma P, Anikhindi SA, Bansal N, Singla V, Khare S, Srivastava A (2020). Is diabetes mellitus associated with mortality and severity of COVID-19? A meta-analysis. Diabetes Metab Syndr.

[15] Erickson N, Mueller J, Shirkov A, Zhang H, Larroy P, Li M, Smola A (2020). AutoGluon-Tabular: robust and accurate AutoML for structured data. https://www.automl.org/wp-content/uploads/2020/07/AutoML_2020_paper_7.pdf.

[16] Che Z, Purushotham S, Cho K, Sontag D, Liu Y (2018). Recurrent Neural Networks for Multivariate Time Series with Missing Values. Sci Rep.

[17] Therneau T, Grambsch P (2000). Modeling Survival Data: Extending the Cox Model.

[18] Cro S, Morris TP, Kenward MG, Carpenter JR (2020). Sensitivity analysis for clinical trials with missing continuous outcome data using controlled multiple imputation: A practical guide. Stat Med.

[19] Buuren SV, Groothuis-Oudshoorn K (2011). mice: Multivariate Imputation by Chained Equations in R. J Stat Soft.

[20] Pan P, Li Y, Xiao Y, Han B, Su L, Su M, Li Y, Zhang S, Jiang D, Chen X, Zhou F, Ma L, Bao P, Xie L (2020). Prognostic Assessment of COVID-19 in the Intensive Care Unit by Machine Learning Methods: Model Development and Validation. J Med Internet Res.

[21] Ko H, Chung H, Kang WS, Park C, Kim DW, Kim SE, Chung CR, Ko RE, Lee H, Seo JH, Choi T, Jaimes R, Kim KW, Lee J (2020). An Artificial Intelligence Model to Predict the Mortality of COVID-19 Patients at Hospital Admission Time Using Routine Blood Samples: Development and Validation of an Ensemble Model. J Med Internet Res.

[22] Abdulaal A, Patel A, Charani E, Denny S, Mughal N, Moore L (2020). Prognostic Modeling of COVID-19 Using Artificial Intelligence in the United Kingdom: Model Development and Validation. J Med Internet Res.

[23] Kim H, Han D, Kim J, Kim D, Ha B, Seog W, Lee Y, Lim D, Hong SO, Park M, Heo J (2020). An Easy-to-Use Machine Learning Model to Predict the Prognosis of Patients With COVID-19: Retrospective Cohort Study. J Med Internet Res.

[24] Bolourani S, Brenner M, Wang P, McGinn T, Hirsch JS, Barnaby D, Zanos TP, Northwell COVID-19 Research Consortium (2021). A Machine Learning Prediction Model of Respiratory Failure Within 48 Hours of Patient Admission for COVID-19: Model Development and Validation. J Med Internet Res.

