# Supplementary material for: COVID-19 Mortality Prediction From Deep Learning in a Large Multistate Electronic Health Record and Laboratory Information System Data Set: Algorithm Development and Validation
Source: J Med Internet Res. 2021 Sep 28;23(9):e30157. doi: 10.2196/30157 (PMC8480399; doi:10.2196/30157)
Supplement: Multimedia Appendix 1 [file jmir_v23i9e30157_app1.docx]

# Multimedia Appendix 1

## Cohort Description

In Figure S1, we visualize the geographic distribution of our cohort within the continental United States. And in Table S1, we provide statistical summaries of each covariate broken down by survival versus death in our cohort.


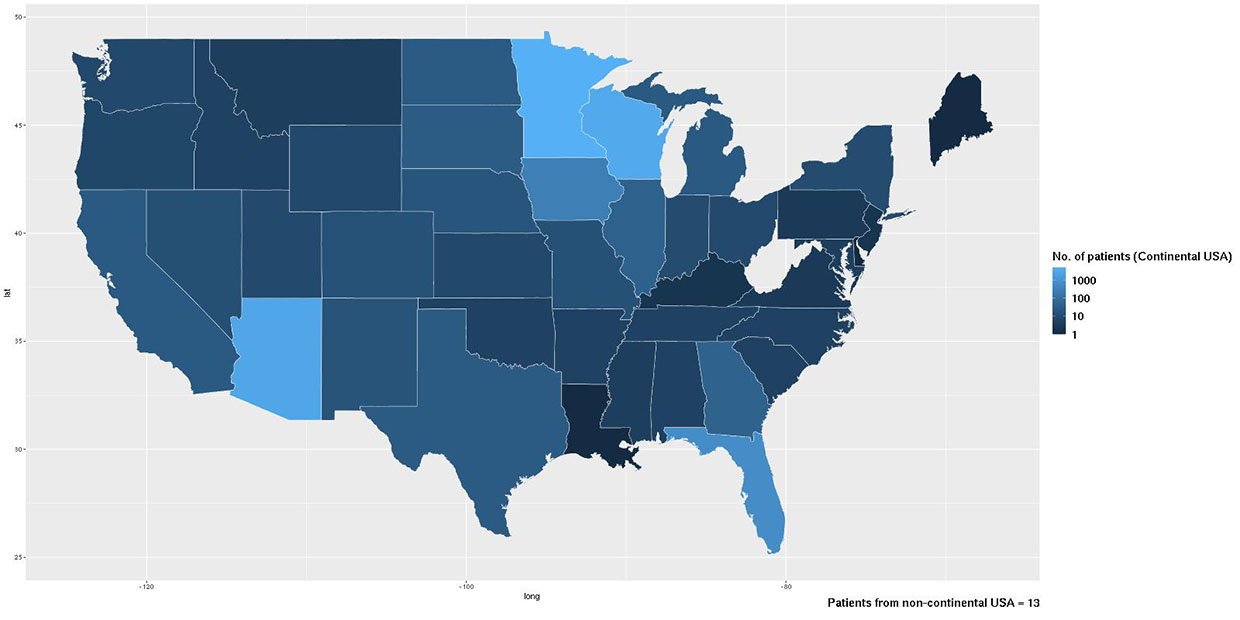


**Figure S1.** The geographic distribution of the Mayo Clinic cohort’s home addresses. Thirteen patients are not represented in the map as they had addresses outside the continental United States.

**Table S1.** Cohort summary with categorical features summarized as number (percentage within group) and numerical features summarized as mean (standard deviation).

|  | **Total** | **Survive** | **Death** |
| --- | --- | --- | --- |
|  | N = 11807 | N = 11263 | N = 544 |
| **Female** | 6198 (52.5%) | 5973 (53%) | 225 (41.4%) |
| **Male** | 5609 (47.5%) | 5290 (47%) | 319 (58.6%) |
| **No chronic kidney disease** | 9776 (82.8%) | 9490 (84.3%) | 286 (52.6%) |
| **Chronic kidney disease** | 2031 (17.2%) | 1773 (15.7%) | 258 (47.4%) |
| **No diabetes mellitus** | 8703 (73.7%) | 8423 (74.8%) | 280 (51.5%) |
| **Diabetes mellitus** | 3104 (26.3%) | 2840 (25.2%) | 264 (48.5%) |
| **Age** | 55.19 (19.94) | 54.20 (19.68) | 75.66 (13.13) |
| **Body mass index** | 30.86 (7.96) | 30.99 (8.02) | 28.57 (6.39) |
| **Weight** | 89.17 (25.08) | 89.49 (25.23) | 82.69 (20.86) |
| **Height** | 169.73 (11.17) | 169.77 (11.22) | 169.05 (10.10) |
| **Charlson** | 66.18 (37.19) | 68.53 (36.23) | 28.69 (31.85) |
| **Basphil count test** | 0.03 (0.03) | 0.03 (0.03) | 0.02 (0.02) |
| **Eosinophil count test** | 0.09 (0.08) | 0.09 (0.08) | Na |
| **Hematocrit test** | 36.15 (6.04) | 36.53 (5.88) | 33.30 (6.54) |
| **Hemoglobin test** | 11.83 (2.18) | 12.01 (2.11) | 10.56 (2.28) |
| **Lymphocyte count test** | 1.35 (4.98) | 1.41 (5.28) | 0.81 (0.62) |
| **Mean corpuscular volume test** | 89.79 (7.25) | 89.76 (6.98) | 89.98 (9.15) |
| **Monocyte count test** | 0.54 (0.30) | 0.55 (0.30) | 0.49 (0.26) |
| **Neutrophil count test** | 4.81 (3.37) | 4.57 (3.14) | 6.62 (4.37) |
| **Platelet count test** | 205.54 (104.10) | 210.35 (94.50) | 171.33 (153.27) |
| **Red cell distribution count test** | 4.05 (0.74) | 4.09 (0.72) | 3.74 (0.84) |
| **Red cell distribution width test** | 14.05 (2.07) | 13.85 (1.91) | 15.40 (2.56) |
| **White blood cell count test** | 6.93 (5.39) | 6.78 (5.46) | 7.95 (4.77) |
| **C-reactive protein test** | 54.59 (56.23) | 51.20 (53.34) | 81.29 (69.74) |
| **D-dimer test** | 1642.09 (4231.08) | 1388.55 (3182.19) | 3690.41 (8728.90) |
| **Ferritin test** | 810.66 (1366.01) | 748.53 (1116.00) | 1240.68 (2442.42) |
| **Interleukin-6 test** | 68.66 (152.18) | 64.88 (149.61) | 91.42 (166.51) |
| **Troponin t test** | 42.14 (155.96) | 35.73 (146.11) | 95.69 (214.75) |
| **Fibrinogen test** | 494.79 (173.78) | 500.24 (171.15) | 464.26 (185.68) |
| **Lactate dehydrogenase test** | 329.31 (608.81) | 301.21 (132.08) | 519.10 (1650.79) |
| **Serum iron test** | 52.36 (57.64) | 53.09 (59.35) | 46.27 (41.42) |
| **Total iron binding capacity test** | 218.20 (74.62) | 220.29 (74.31) | 200.86 (76.69) |
| **Percentage iron saturation test** | 24.25 (21.22) | 24.09 (21.24) | 25.59 (21.50) |
| **Transferrin test** | 184.90 (63.25) | 186.69 (62.99) | 170.14 (64.95) |
| **Bilirubin test** | 0.52 (0.70) | 0.48 (0.33) | 0.83 (1.81) |
| **Albumin test** | 3.45 (0.55) | 3.50 (0.53) | 3.07 (0.59) |
| **Bicarbonate test** | 23.77 (3.36) | 23.96 (3.23) | 22.28 (4.00) |
| **Blood urea nitrogen test** | 24.07 (17.11) | 22.38 (15.37) | 37.55 (23.36) |
| **Creatinine test** | 1.38 (1.44) | 1.33 (1.44) | 1.77 (1.44) |
| **Potassium test** | 4.21 (0.51) | 4.22 (0.50) | 4.16 (0.53) |
| **Sodium test** | 138.09 (4.11) | 138.08 (3.91) | 138.15 (5.51) |
| **Chloride test** | 102.07 (4.81) | 101.90 (4.54) | 103.38 (6.42) |
| **Glucose test** | 134.75 (55.09) | 134.20 (55.80) | 138.93 (49.55) |
| **Calcium test** | 8.52 (0.59) | 8.55 (0.58) | 8.28 (0.63) |
| **Maximum blood pressure systole** | 135.35 (24.61) | 135.73 (24.02) | 130.84 (30.38) |
| **Minimum blood pressure systole** | 119.54 (15.99) | 119.89 (15.90) | 115.42 (16.56) |
| **Maximum blood pressure diastole** | 82.45 (11.16) | 82.52 (11.02) | 81.62 (12.64) |
| **Minimum blood pressure diastole** | 71.47 (17.72) | 71.80 (17.12) | 67.57 (23.31) |
| **Maximum temperature** | 98.58 (1.19) | 98.57 (1.19) | 98.71 (1.18) |
| **Minimum temperature** | 97.74 (2.00) | 97.77 (2.02) | 97.42 (1.61) |
| **Maximum pulse** | 88.88 (18.34) | 88.43 (18.01) | 94.04 (21.19) |
| **Minimum pulse** | 70.34 (15.64) | 70.77 (15.59) | 65.33 (15.26) |
| **Maximum respiratory rate** | 22.37 (7.43) | 21.94 (7.08) | 27.20 (9.31) |
| **Minimum respiratory rate** | 15.98 (3.41) | 16.03 (3.22) | 15.48 (5.05) |
| **Maximum oxygen saturation** | 97.67 (2.02) | 97.67 (1.93) | 97.59 (2.86) |
| **Minimum oxygen saturation** | 92.37 (5.83) | 92.86 (4.97) | 86.80 (10.34) |
| **Not ventilated** | 8931 (75.6%) | 8727 (77.5%) | 204 (37.5%) |
| **Ventilated** | 2876 (24.4%) | 2536 (22.5%) | 340 (62.5%) |
| **Survive past 21days** | 11454 (97%) | 11263 (100%) | 191 (35.1%) |
| **Death within 21days** | 353 (3%) | 0 (0%) | 353 (64.9%) |

### Error Analysis

To supplement the findings of our survival analysis, we embark here to study the false positives and negatives for the selected threshold on our ROC curve. To this end, we have examined the ten variables of highest importance to our model, stratified by the categories of our confusion matrix, i.e., true negative (TN), false positive (FP), false negative (FN), and true positive (TP). Figures S2-S9 represent the continuous variables whereas Table S2 captures the data for CKD status. While CKD conflates missingness with lack of the condition, the continuous variables do not suffer from this limitation and therefore the percentage of patients with missing values in each confusion matrix category are included. When multiple time points were available for a given data type, only the most recent observation was included, in order to ensure only a single data point per patient.

Examination of the described results uncovers notable trends in the data. Firstly, the false negatives tend to have higher rates of missing data. Missing data not only hampers our model’s ability to accurately gauge the outcome status of these patients, but it also indicates that–at least in the first 72 hours of confirmed infection the clinical suspicion on these patients was low. Compounding the issue of missing data is the fact that the more readily available metrics such as age trend in the direction of favorable prognoses for these false negative patients. For instance, the FN cohort is younger than the TP and FP predicted by GRU-D to be at high risk. Likewise, the Charlson estimated 10-year survival rate differs across our groups in a predictable manner, with predicted risk by GRU-D being anticorrelated with 10-year survival estimates, and our false negatives have correspondingly higher Charlson survival estimates. Interestingly, GRU-D is able to correctly classify both negatives and positives at the high and low end of the Charlson scale, indicating that GRU-D is not a simple recapitulation of Charlson predictions. Furthermore, the univariate trends between TP versus TN in the data presented are increased Age, FERR, D-DIMER; decreased Charlson 10-year survival rate, weight, FIBTP, IRON and MinSpO2. The FN and FP tended to be intermediate between these values, with FN more closely resembling TN and FP more closely resembling TP as one would expect based on the behavior of a prediction algorithm.

Finally, we note that no univariate marker appears to cleanly discern the true labels (i.e., red versus green in the violin plots for death versus survival, respectively). Furthermore, the reductions in AUROC from dropping univariate markers in Figure 3 were relatively small, indicating both redundancy in the information represented by these features and a general importance of multivariate context. Figure S9, for instance shows no stratification of the individual groups, but this is likely due to the fact that covariates including sex, age and height will play a necessary role in con- textualizing weight from a clinical prognostication point of view. Ultimately however, the univariate trends displayed here provide a good first-order approximation to understanding the risk factors for mortality in COVID-19, which coincide with known biology.


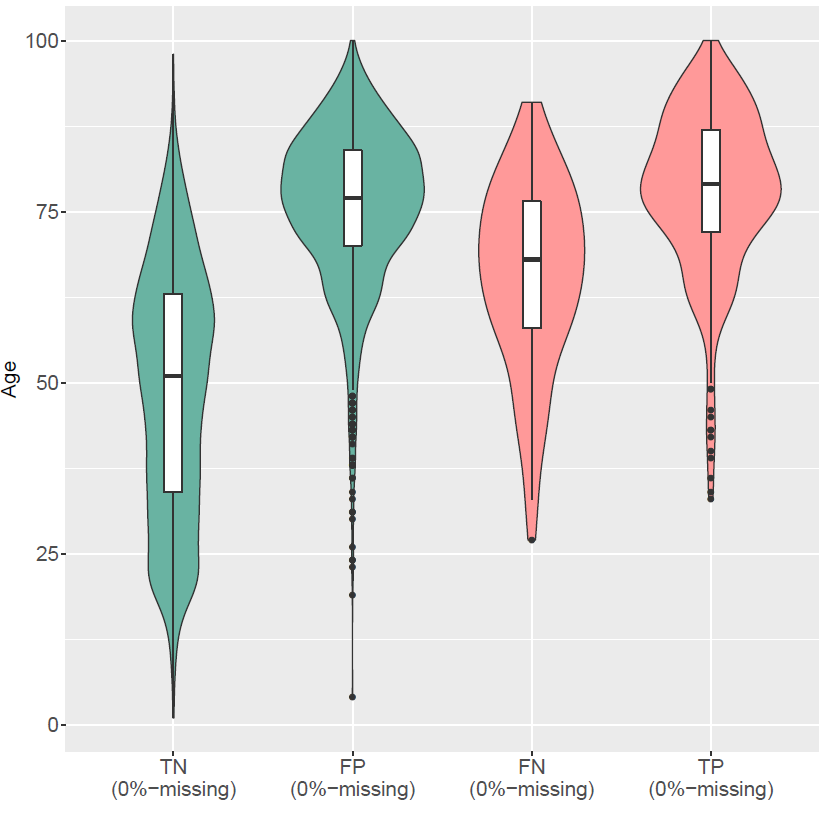


**Figure S2.** Violin plot of age (years) versus confusion matrix categorization: true negative (TN), false positive (FP), false negative (FN), and true positive (TP). Color indicates true state of the patient as survivor (green) or non-survivor (red). Percentage of missing values in each category is indicated at the base of the plot.


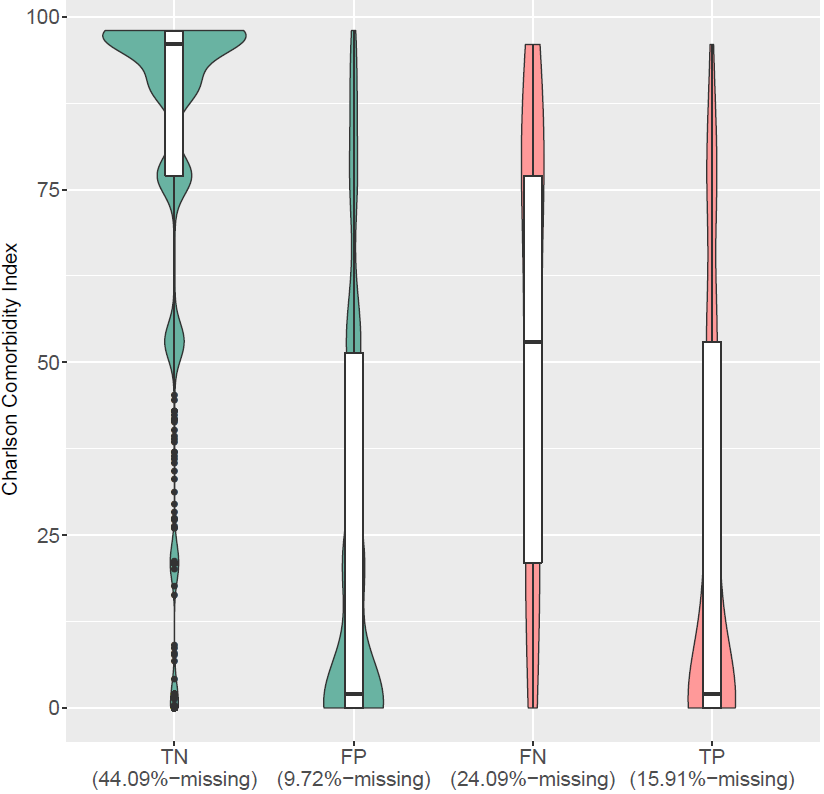


**Figure S3.** Violin plot of Charlson Comorbidity Index (10-year survival probability) versus confusion matrix categorization: true negative (TN), false positive (FP), false negative (FN), and true positive (TP). Color indicates true state of the patient as survivor (green) or non-survivor (red). Percentage of missing values in each category is indicated at the base of the plot.


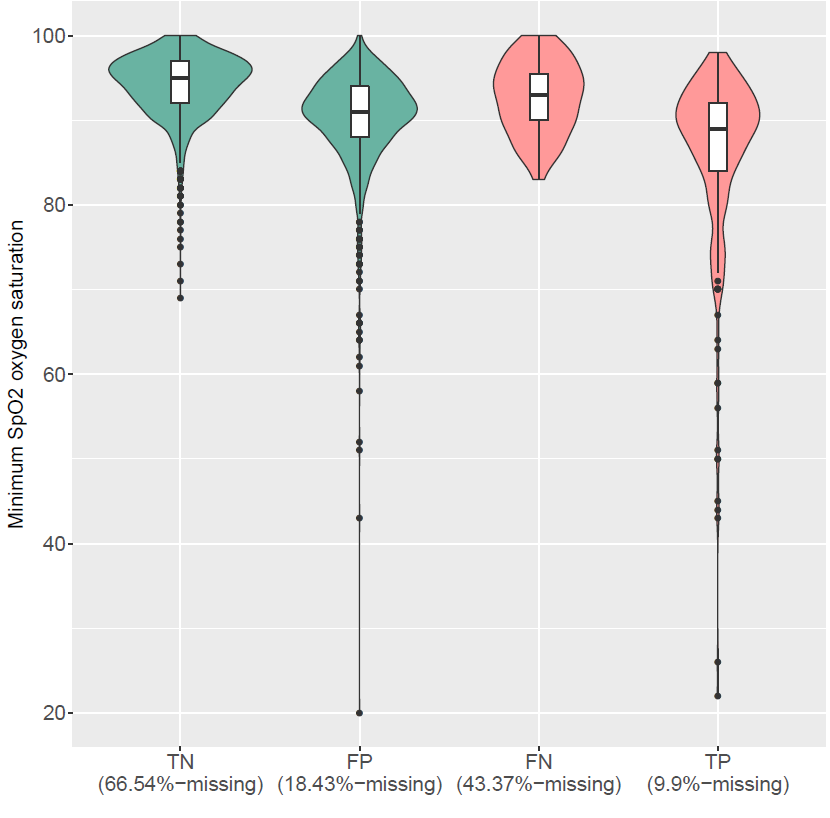


**Figure S4.** Violin plot of MinSpO2 (%) versus confusion matrix categorization: true negative (TN), false positive (FP), false negative (FN), and true positive (TP). Color indicates true state of the patient as survivor (green) or non-survivor (red). Percentage of missing values in each category is indicated at the base of the plot

**Figure S5.** Violin plot of FIBTP (mg/dL) versus confusion matrix categorization: true negative (TN), false positive (FP), false negative (FN), and true positive (TP). Color indicates true state of the patient as survivor (green) or non-survivor (red). Percentage of missing values in each category is indicated at the base of the plot.

**Figure S6.** Violin plot of IRON (mg/dL) versus confusion matrix categorization: true negative (TN), false positive (FP), false negative (FN), and true positive (TP). Color indicates true state of the patient as survivor (green) or non-survivor (red). Percentage of missing values in each category is indicated at the base of the plot.

**Figure S7.** Violin plot of FERR (mg/L) versus confusion matrix categorization: true negative (TN), false positive (FP), false negative (FN), and true positive (TP). Color indicates true state of the patient as survivor (green) or non-survivor (red). Percentage of missing values in each category is indicated at the base of the plot.

**Figure S8.** Violin plot of the D-DIMER (ng/mL) versus confusion matrix categorization: true negative (TN), false positive (FP), false negative (FN), and true positive (TP). Color indicates true state of the patient as survivor (green) or non-survivor (red). Percentage of missing values in each category is indicated at the base of the plot.


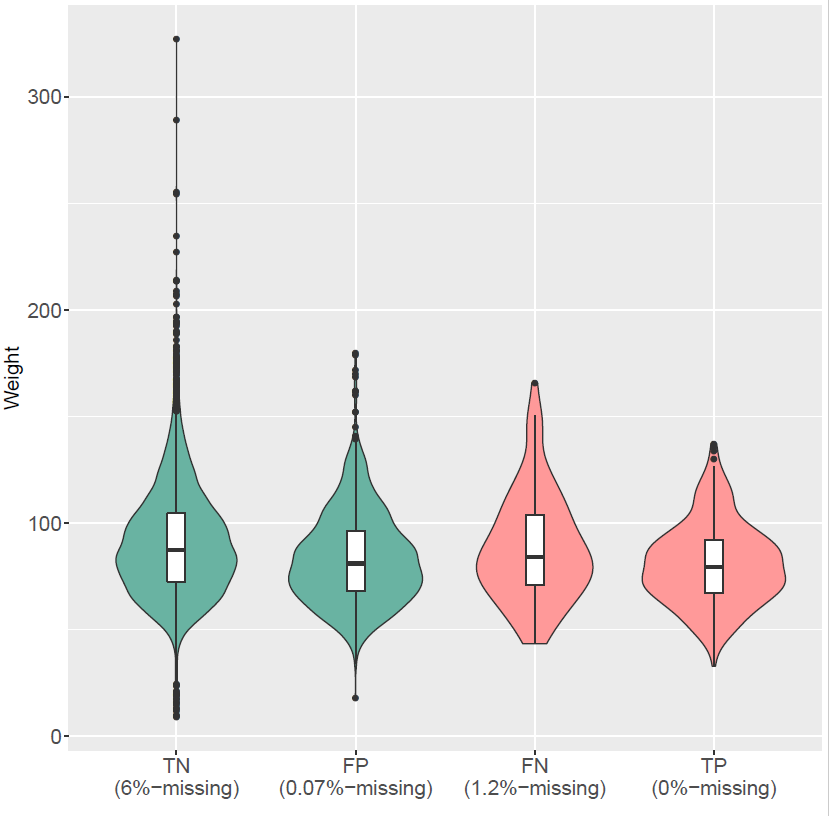


**Figure S9.** Violin plot of weight (kg) versus confusion matrix categorization: true negative (TN), false positive (FP), false negative (FN), and true positive (TP). Color indicates true state of the patient as survivor (green) or non-survivor (red). Percentage of missing values in each category is indicated at the base of the plot.

**Table S2:** Chronic Kidney disease Error Analysis

|  | TN | FP | FN | TP |
| --- | --- | --- | --- | --- |
|  |  |  |  |  |
| No CKD | 7073 | 663 | 68 | 146 |
| CKD | 568 | 715 | 15 | 187 |

**Table S3:** Serology Error Analysis

|  | TN | FP | FN | TP |
| --- | --- | --- | --- | --- |
|  |  |  |  |  |
| Negative | 305 | 91 | 0 | 34 |
| Positive | 155 | 33 | 4 | 8 |
