# Supplementary material for: COVID-19 Mortality Prediction From Deep Learning in a Large Multistate Electronic Health Record and Laboratory Information System Data Set: Algorithm Development and Validation
Source: J Med Internet Res. 2021 Sep 28;23(9):e30157. doi: 10.2196/30157 (PMC8480399; doi:10.2196/30157)
Supplement: Multimedia Appendix 2 [file jmir_v23i9e30157_app2.docx]

# Multimedia Appendix 2

### Monte Carlo missing not-at-random simulation

**Figure S1.** The results of 1000 Monte Carlo samples from the MNAR example. Red dots indicate aberrant labs and green indicate normal labs that will have one of their values censored by a Bernoulli trial.

Here we construct a simple model to demonstrate the power of MNAR data in classification tasks, and the challenge they pose in imputation strategies. Consider the random variables 𝑋_1_,𝑋_2_,𝑌, where we assume 𝑌 is the binary class label for severe 𝑌 = 1 or non-severe disease 𝑌 = 0, and 𝑋_1_ and 𝑋_2_ are two lab values that are zero-mean jointly Gaussian features with covariance matrix

∑ =

Suppose normal labs have 𝑋1 · 𝑋2 < 0 and patients with abnormal labs (i.e., 𝑋1 · 𝑋2 ≥ 0) are the only individuals going to have severe disease. Now suppose the physician is randomly (i.e., chosen by Bernoulli trial) provided a single lab value, but has perfect clinical judgement only orders the second missing clinical tests to confirm a patient is going to have severe disease; those without severe disease will thus only have a single lab value measured. These missing values will be MNAR. The class variable 𝑌 = 1 whenever the features are in the lower left or upper right quadrant of the feature space (i.e., 𝑋1 ≤ 0 ≥ 𝑋2 or 𝑋2 ≥ 0 ≤ 𝑋1), and 𝑌 = 0 otherwise; see Figure S1 for an example Monte Carlo dataset. Clearly, in the fully observed dataset a simple decision tree can perfectly classify this data, but here whenever 𝑌 = 0 one of the features is randomly selected to be missing. The common missing completely at random (MCAR) strategy of “complete case analysis” wherein one simply removes all rows with missing data will clearly fail completely here since no 𝑌 = 0 data would make its way into the training set. Likewise, treating the data as MCAR and imputing based on the known mean of zero will result in all normal labs being in the abnormal ranges (and the more common strategy of using an empirical mean or median clearly only adds small noise to this imputation). More sophisticated missing at random (MAR) strategies such include using a KNN to find the nearest point in the reduced feature space to fill the missing values, or using the more sophisticated statistical imputation tools such as the multiple imputation by chained equations (MICE) method implemented in the mice library in R [24] to randomly fill in missing values while accounting for observed variables and to perform model averaging over the many randomly imputed datasets. In this classification context all such strategies will fail, and the only successful techniques will employ added features for missingness indicator variables to allow the classifier to explicitly model missing data patterns. We demonstrate these facts by employing the above techniques with a decision tree in our Monte Carlo samples, using an 80/20 train/test split.

**Figure S2.** One sample of MICE imputation on the MC samples from Figure S1.

In the fully observed data, as expected the decision tree performs very well producing the confusion matrix (rows are true label, columns are predicted)

In the complete case analysis, training is not even possible since all 𝑌 = 0 labels are thrown out from the analysis, and thus at best we are left with a dummy classifier always predicting class 𝑌 = 1, resulting in the confusion matrix

If we consider the state-of-the-art MICE method, allowing it the benefit of using the class label in its multiple imputations modeling and filling in the entire test/train dataset (a data leakage problem giving undue advantage to the method), we still find the imputation fails and gives poor performance even with model averaging, resulting in the confusion matrix

The failure of this method can be seen clearly when we plot one of the imputed datasets produced by this method in Figure S2.

Finally, if we use a decision tree on only the missingness indicators, we achieve perfect performance (exceeding even the fully observed data)

Clearly this example is overly simplified and taken to an extreme in terms of the coupling of missingness to class labels, but it illustrates the salient points surrounding MNAR in classification tasks when such coupling exists. In our real data experiments, we have verified that this coupling exists (albeit to a lesser extent than this toy example) with lab value ordering. GRU-D is a neural network architecture that has been designed around the notion of leveraging MNAR in multivariate time series classification tasks and the authors plan to invest further research effort in this direction.
